# A High Compliment to Dr. Q. C. Smith

**Published:** 1886-07

**Authors:** 


A HIGH COMPLIMENT TO A DISTINGUISHED TEXAS PHYSICIAN.—Dr. Q. C. Smith, of Austin, Texas, has been made a Fellow of the Society of Science, Letters and Art of London, and has received his certificate, with the compliments of, and an autographic letter from the Hon. President, Sir Henry V. Goold—Bart.

